# Do Singing Rock Hyraxes Exploit Conspecific Calls to Gain Attention?

**DOI:** 10.1371/journal.pone.0028612

**Published:** 2011-12-06

**Authors:** Amiyaal Ilany, Adi Barocas, Lee Koren, Michael Kam, Eli Geffen

**Affiliations:** 1 Department of Zoology, Tel Aviv University, Tel Aviv, Israel; 2 Program in Ecology and Department of Zoology and Physiology, University of Wyoming, Laramie, Wyoming, United States of America; 3 Department of Comparative Biology and Experimental Medicine, University of Calgary, Calgary, AB, Canada; 4 Desert Animal Adaptations and Husbandry, Wyler Department of Dryland Agriculture, The Jacob Blaustein Institutes for Desert Research, Ben-Gurion University of the Negev, Beer-Sheva, Israel; University of Sussex, United Kingdom

## Abstract

Signal detection theory predicts that signals directed at distant or busy receivers in noisy backgrounds will begin with an alert component, in order to draw attention. Instead of an alert component, however, animals could get the same effect by using an external stimulus. Here we combined observations of free-living rock hyraxes (*Procavia capensis*) with playback experiments to elucidate the circumstances under which males begin singing. We show that males sing following hyrax pup screams, which elicit a strong response from hyraxes within hearing distance, which are potential receivers. We hypothesize that singers enhance their singing display by exploiting the rarely emitted pup screams. To our knowledge, our findings are the first indication that animals may enhance signal reception by exploiting conspecifics' signals and the differential attention to these signals. We suggest that the utilization of external stimuli by signalers may be widespread, as an adaptive strategy for communication in complex environments.

## Introduction

Social communication requires that receivers are able to detect relevant signals and pay attention to significant details in complex signals, in order to decode information. However, environmental noise and receivers' activity may impair communication efforts [1]–[4]. Specifically, the effectiveness of acoustic communication may suffer from ambient noise and lack of attention by potential receivers [1], [5].

To enhance receiver attention to signals, signal detection theory predicts the use of alert components. These are aimed at drawing the attention of potential receivers, thus overcoming environmental noise before delivering the main part of the signal [3]. The need for alert signals increases with the complexity of the main signal, or of the information encoded in it, because sub-optimal environmental conditions weaken complex signals more than simple ones [3]. Alert signals may enhance any signal, whether communicated in the visual, acoustic or chemical mode [3], although few studies have elucidated their use [1], [3].

Instead of adding an alert component to their signals, animals may also enhance signal reception through external alert stimuli. The signaler could draw the attention of potential receivers by utilizing a conspicuous external signal. Effective external signals should theoretically be rare, in order to elicit a stronger response from receivers. We are aware of only one example that suggests the use of external signals to elicit attention to the main signal, in the splendid fairy-wren [6], [7].

The rock hyrax (*Procavia capensis*) is a social mammal that lives in mixed-sex groups, consisting in our study area of several males (one mature immigrant resident and several natal late dispersers) and five to 20 females with their pups. Females constitute the core of the group while resident males remain with a group for up to four years [8]. Males contribute a small proportion of caring for group pups by babysitting and guarding [9], while females share daily parenting responsibilities. Breeding is seasonal and synchronized [10] and females mate with multiple males, masking paternity [11]. Adolescent males (i.e., 17–24 months old) are forced to disperse [12] and they live on the periphery of colonies [13].

Acoustic communication constitutes the most widely used means of information transfer among rock hyraxes [14]. Both males and females produce loud repetitive warning trills, while adult male hyraxes also engage throughout most of the year in rich and complex vocalizing behavior we term “singing” [13]. Singing in the rock hyrax may be related to sexual advertisement since it abruptly decreases for a few months following the mating period [13]. Rock hyrax male songs are complex signals that encode multiple types of information, such as identity, age, weight, size, social rank and hormonal status of the singer [15]. Males sing in different contexts. Our observations suggested that male hyraxes tend to sing in response to other males' songs and to the screams that pups give when they are handled for marking. Pup screams are presumably produced only in the presence of imminent danger and thus may elicit a strong attention response from all hyraxes in the vicinity. Consequently, they provide a suitable background to test the hypothesis that males use pup screams as external alert signals, providing them with an opportunity for self-advertisement. We played back recordings of two different signals produced by hyraxes: foreign male songs and pup screams, to male and female hyraxes and recorded their behavioral and vocal responses. We predicted a stronger response to pup screams than to male songs by both sexes. If pup screams are treated as distress call, we predict male response in a form of approaching the screaming pup or looking at its direction. On the other hand, if pup screams are viewed as an opportunity for self-advertisement, we predict males to respond by singing. Alternatively, since males provide little parental care, they may ignore pup screams and respond more strongly to male songs.

## Methods

### Ethical Statement

Since the rock hyrax is protected under the Israeli law, all relevant permits are given by the Nature and Parks Authority and not by the relevant university. Permits for capturing, marking and handling the hyrax were issued and reviewed annually by the Israeli Nature and Parks Authority (permit numbers: 2000/8871, 2001/8871, 2002/14674, 2003/14674, 2004/17687, 2005/17687, 2007/27210, 2008/31138, 2009/32871).

### Study Site and Population

Fieldwork took place in two deep gorges, David and Arugot, in the Ein Gedi Nature Reserve (31°28′N, 35°24′E), Israel. In each gorge we monitored two mixed-sex groups, and the bachelor males in their peripheries.

Live box traps (Tomahawk Live Trap Co, Tomahawk, WI, USA) were set before first light (approximately 90 min before dawn) with kohlrabi and cabbage as bait, and operated until late morning, with inspections every two hours. Traps were placed in shaded locations and were not vulnerable to predators. No water was supplied in the traps as hyraxes are rarely observed drinking. Except for pups, newly trapped animals were anaesthetized with ketamine hydrochloride (0.1 ml/kg intramuscular injection), weighed, measured, photographed, and individually marked using small (1 cm) cylindrical subcutaneous glass transponders (DataMars SA, Bedano-Lugano, Switzerland) that were inserted into the neck region and remained there for the duration of this study. No adverse effects of the transponders were observed during 11 years of study. All measurements were recorded in situ, and the animals were returned to the traps for full recovery (3 h), and thereafter released at the capture site. Pups, which were 2–5 months old, were handled for one minute to record their screams. All animals were handled and marked in shaded places to prevent heat shock. Animals resumed full normal activity following their release.

### Vocalization and Behavioral Recording

**Figure 1:**
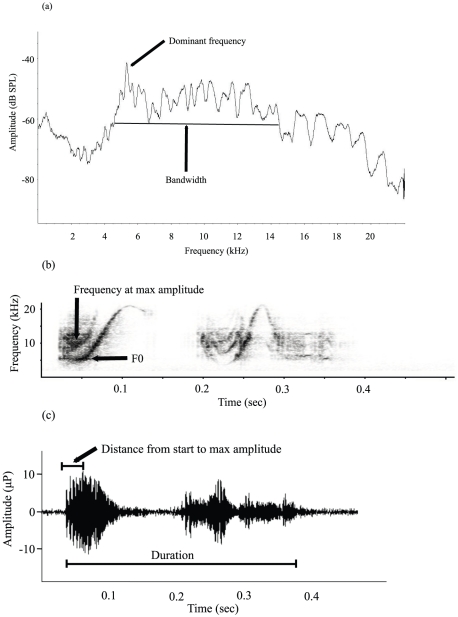
Acoustic parameters of rock hyrax pup screams. (a) A spectrum of a scream with two sub-elements. The amplitude is shown as a function of frequency. (b) A spectrogram showing frequency as a function of time. (c) A waveform showing the amplitude as a function of time.

When hand-held, most hyrax pups produce loud screams (Fig. 1). Nine screaming pups were recorded from a distance of two meters, with a Sennheiser ME 67 shotgun microphone (frequency response 50–20,000 Hz ±2.5 dB) powered by a Sennheiser K6 module, covered with a Sennheiser MZW70-1 blimp windscreen (Sennheiser Electronic GmbH & Co. K. G., Wedemark, Germany). The microphone was hand-held or on a tripod, using an MZS20-1 shock-mount with a pistol grip. Screams were recorded in mono (Tascam HD-P2 digital audio recorder; TASCAM Corporation, Montebello, CA, USA), with a sampling frequency of 48 kHz and a sampling width of 24 bits.

During nine field seasons (March-August 2000–2005, 2007–2009) we observed hyrax behavior almost daily, using 10×42 binoculars and a Celestron C5 telescope (x75 magnification; Celestron, Torrance, CA, USA). When singing was observed, the singer's identity and singing context were recorded. The songs were recorded using the same equipment from a distance of 10–50 m [11].

### Playback Experiments

Recordings were normalized to 75 ± 5 dB and played via a FoxPro FX3 speaker (FOXPRO Inc., Lewistown, PA, USA) using a remote control (FoxPro TX5-LR). In each experimental session the remote-controlled speaker was mounted before dawn in a location that hyraxes frequent. When a hyrax came within a distance of 10–30 m from the speaker, either a pup scream or a male song was played. Sounds recorded in David gorge were played back to Arugot hyraxes and vice versa. Each hyrax (55 males, 115 females) participated in our playback experiments only once. We verified that other hyraxes were not present near the speaker, so that the tested individual would not perceive the playback as coming from any specific visible hyrax. We noted all hyrax responses. A singing response was noted if a male started singing within 30 seconds of the playback. To minimize pseudo-replications, we used nine independent (i.e. from different individuals) recordings of each call type – six from the Arugot gorge and three from the David gorge. No more than two experiments were performed on the same day, thus preventing acclimatization by nearby hyraxes. A two-tailed Fisher's exact test was used to analyze the playback experiment results.

## Results

**Figure 2:**
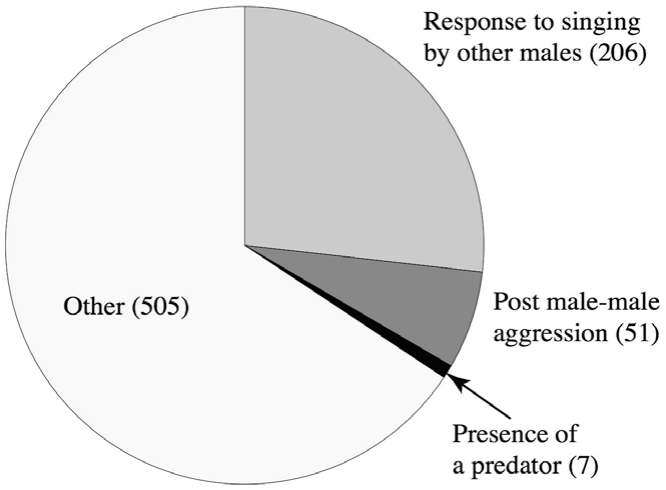
The distribution of events inducing male singing in natural circumstances over 11 years. No naturally recorded singing events occurred in response to pup screams.

Out of 769 naturally recorded male singing events spanning nine field seasons (excluding the playback experiments), 206 events (26.8%) were in response to other males singing, and 51 (6.6%) followed an agonistic interaction with another male. Seven (0.9%) of the singing events were in the presence of predators (i.e. leopards, wolves, striped hyenas and foxes; out of 60 predator encounters). We could not determine the trigger for the remaining 505 singing events (65.7%), but they were clearly not directly related to male-male aggression (Fig. 2). Only four cases of females singing were observed during nine years and approximately 3500 hours of hyrax observations at the Ein Gedi Nature Reserve.

**Figure 3:**
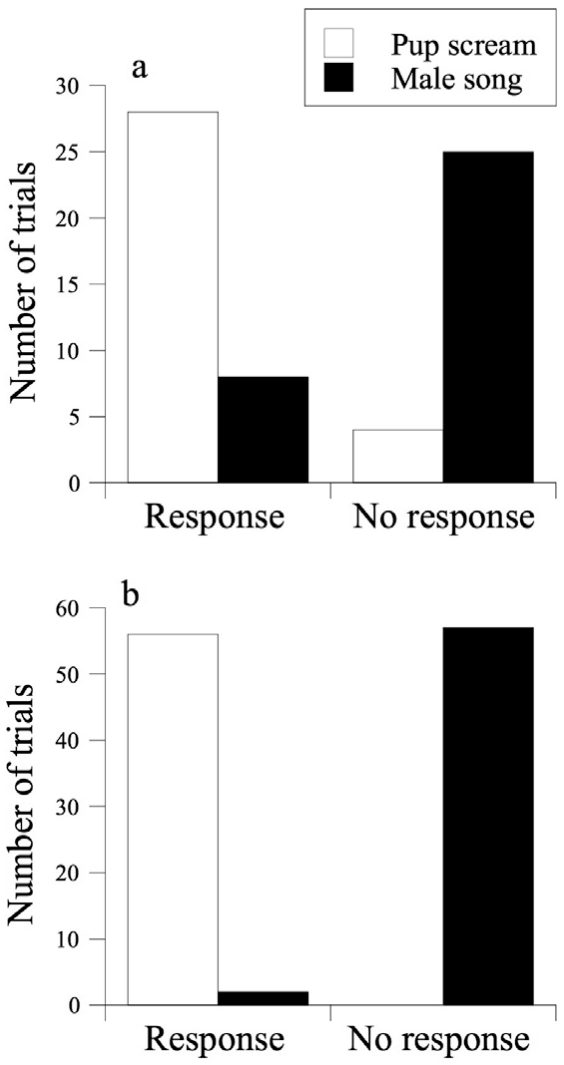
Playback experiments results. (a) The number of times males responded to playbacks of pup screams or foreign males' songs by singing. White bars denote pup screams; black bars denote male songs. The response to pup scream was significantly stronger (Fisher's exact test, *P* < 0.0001). (b) The number of times females responded to playbacks of pup screams or foreign males' songs by looking in the direction of the speaker. White bars denote pup screams; black bars denote male songs. The response to pup scream was significantly stronger (Fisher's exact test, *P* < 0.0001).

During playback experiments, 84.3% of the males (n = 32) responded to pup screams by singing, whereas only 21.2% of the males (n = 33) responded to the male songs by singing. No males moved towards the speaker. Overall, male hyraxes responded significantly more to pup screams than to unknown male songs (Fisher's exact test, *P* < 0.0001, Fig. 3a).

In playbacks to females, 100% of the females (n = 56) responded to pup screams by looking in the direction of the speaker for at least one minute, and six of them ran towards it. Only 3.4% of the females (n = 59) looked at the speaker when male songs were broadcasted (Fisher's exact test, *P* < 0.0001, Fig. 3b).

## Discussion

Under natural conditions, we found that male hyraxes sang in a range of circumstances, for example, subsequent to other males' songs and escalated male-male conflicts, but rarely in the presence of predators. Our observations of male singing in response to other males and after agonistic interactions, suggest that singing plays a role in intrasexual communication. Singing may also advertise male quality to females because singing significantly decreases after the mating season [13]. Singing is heard from a distance of hundreds of meters, but potential listeners may receive a degraded signal due to environmental noise such as waterfalls, wind, and bird songs. Potential listeners may also be busy foraging, resting or babysitting and pay little attention to male songs. Our data suggests that males may therefore exploit specific circumstances that hyraxes are alert in order then to sing and advertise themselves. In our study site, hyraxes are usually observed from a distance of 50–100 meters. Our ability to hear naturally occurring pup screams is thus limited. Consequently, we cannot reliably assess how frequent pup screams are, and some of the male songs we recorded may have been a response to pup screams we failed to detect.

Pup screams were found to be highly evocative in the yellow-bellied marmot (*Marmota flaviventris*), eliciting a strong response of increased vigilance from both males and females [16]. Our experiments showed that pup screams triggered males to sing much more than other males' songs did. Our experience of trapping and marking pups revealed that pups do not scream before being actually handled, suggesting that pup screams are used only under extreme danger. Indeed, when a pup screamed all hyraxes in the vicinity stopped their activity and look attentively in the direction of the pup, and some adult females ran towards it. Consequently, a male hyrax that sang during this event, may have exploited the screams as alert signals to advertise itself, enhancing the reception of its song messages. The singing response to pup screams is not an artifact of our long-term research or site dependent. Males in 20 other locations across Israel, where no research is routinely performed, responded to pup scream playbacks by singing (unpublished data).

The response of hyraxes to pup screams resembles the way splendid fairy-wrens (*Malurus splendens*) sing after gray butcherbirds (*Cracticus torquatus*) vocalize, which was found to draw the attention of potential receivers [7]. Butcherbirds prey on splendid fairy-wrens, and their vocalizations were shown to serve as an alert signal, enhancing female fairy-wrens' response to male signals following the predator vocalization. Another example of attention grabbing is the use of different roar types by calling red deer (*Cervus elaphus*), where male ‘harsh roars’ were shown to alert females and draw their attention to the ‘common roars’ that follow [17]. In this case, the alert signal is internal rather than external, produced by the calling male itself. Calls that included the alerting ‘harsh roars’ midway through the vocal sequence drew increased attention from female receivers and maintained attention to subsequent calls [17]. Attention grabbing by using harsh sounding calls has also been found in meerkats (*Suricata suricatta*) [18] and chimpanzees (*Pan troglodytes*) [19]. In these two examples, individuals modify their calls to match the level of urgency due to predators or conspecific aggression, but their calls do not serve as alert signals to subsequent calls. The usage of alert signals in a different modality was found in the visual display of the yellow-chinned anole (*Anolis gundlachi*). Males of this species were shown to add an alert component to their visual display when communicating to distant receivers in situations of poor visibility, thus enhancing signal detection [3]. The aforementioned examples suggest that animals use a wide range of internal or external signals to enhance signal reception. Our results imply a similar case, although this one is novel in that males exploit conspecifics’ signals, and not their own signals or the signals of another species, to serve as alert signals.

Singing in the presence of predators may also constitute a ‘handicap’ [20] advertising the singer's quality, as it exposes itself to potential predation risk. Under this hypothesis, only high quality males should be able to risk singing in the presence of predators. However, our observations showed that when predators were silent, only a few hyraxes sang (11.7% of predator encounters). This suggests that singing may only be advantageous when other hyraxes are aware of the possible risk, such as following pup screams or predator sounds. Interestingly, splendid fairy-wrens were found to call only in response to predator calls, but like hyrax, not in the presence of silent predators [21], supporting this idea.

Male hyraxes seldom behave in a manner that can be classified as parental care [9]. Could singing in response to pup screams constitute parental care? The situation in which males tend to sing and the structure of songs suggest they are more likely to serve in self-advertisement. Yet, if infanticide is possibly a threat to pups, then male singing following pup screams may serve to deter rival males from attacking their pups. As females mate with multiple males in the mating season [8], and males may not identify their own pups by their screams, males may employ a “sing when you hear any pup scream” strategy. Singing may inform the attacker of the presence of a protective male, communicating its strength, even if the singer is not near the attacked pup. However, if this was the case, why would males select to sing rather than attack an aggressive male? As pups only scream when in imminent danger, singing may be ‘too little, too late’ to save a pup solely through conveying general information about the singer. Furthermore, our field observations suggest that infanticide in rock hyraxes is a rare event (only one observed case in 11 years), making this notion of vocal warning unlikely. The repetitive pattern of the songs further suggests that they are intended to display the singer's quality, rather than elicit an immediate response from a potential attacker [22].

Our results suggest that male hyraxes adaptively use pups' distress to enhance the impact of their self-advertisement. The only response we observed to male singing was counter-singing by neighboring males. Unfortunately, we cannot detect a behavioral reaction in female hyraxes to male singing under natural conditions or playbacks. Male singers appear to accurately and actively exploit the single most attention-provoking signal in the hyrax vocal repertoire, in order to draw the attention of all potential audiences, both rival males and potential female mates, to set the stage for their own vocal performance. Our data further supports the hypothesis that signaling in an event that alerts conspecifics may be widespread across species and modalities [7]. We suggest that this behavior constitutes a type of sensory exploitation [23] for communicating in complex environments and competitive social situations.
